# Mast Cells as Regulators of T Cell Responses

**DOI:** 10.3389/fimmu.2015.00394

**Published:** 2015-08-07

**Authors:** Silvia Bulfone-Paus, Rajia Bahri

**Affiliations:** ^1^Manchester Collaborative Centre for Inflammation Research (MCCIR), Institute of Inflammation and Repair, University of Manchester, Manchester, UK

**Keywords:** mast cells, CD4 T cell, CD8 T cell, Treg cells, adaptive immunity

## Abstract

Mast cells (MCs) are recognized to participate in the regulation of innate and adaptive immune responses. Owing to their strategic location at the host–environment interface, they control tissue homeostasis and are key cells for starting early host defense against intruders. Upon degranulation induced, e.g., by immunoglobulin E (IgE) and allergen-mediated engagement of the high-affinity IgE receptor, complement or certain neuropeptide receptors, MCs release a wide variety of preformed and newly synthesized products including proteases, lipid mediators, and many cytokines, chemokines, and growth factors. Interestingly, increasing evidence suggests a regulatory role for MCs in inflammatory diseases via the regulation of T cell activities. Furthermore, rather than only serving as effector cells, MCs are now recognized to induce T cell activation, recruitment, proliferation, and cytokine secretion in an antigen-dependent manner and to impact on regulatory T cells. This review synthesizes recent developments in MC–T cell interactions, discusses their biological and clinical relevance, and explores recent controversies in this field of MC research.

## Introduction

Mast cells (MCs) are among the most malleable and rapidly responding cells of the immune system. Within seconds of activation, they release a multitude of preformed biologically active products, followed by marked changes in cytoplasmic composition and volume that enable reconstitution of their morphology and cell content within hours (1, 2). Counterintuitively, this cell-regenerative phase coincides with a striking wave of mediator synthesis and secretion. Therefore, tissue-resident MCs have the potential to strongly shape their tissue microenvironment and direct cell–cell interactions and immune cell responses even while running through a reconstitution phase, during which they are relatively “refractory” to external stimuli.

Derived from either hematopoietic precursors or local, tissue-resident progenitors, mature MCs represent a heterogeneous collective of long lived, granulated cells located in essentially all tissues, which increase in number upon proliferation or increased recruitment, survival, and/or maturation of MC progenitors (1–3). They are particularly abundant at barrier sites, such as the skin, lung, and gut, and play an important role in defense against, and clearance of various pathogens (4, 5).

While the involvement of MCs in allergic/inflammatory reactions triggered by the crosslinking of FcεRI-bound immunoglobulin E (IgE) by antigen has been characterized in detail (6), the extent of MC function in autoimmune diseases is less well understood (7, 8). Upon activation, MCs release a plethora of mediators, including growth factors, cytokines, and chemokines (e.g., IL-1, IL-6, IL-8, IL-10, TNFα, VEGF, TGFβ, CCL2-4) as well as pro-inflammatory lipid mediators, such as prostaglandins and leukotrienes. However, MCs are mostly known for the ability to degranulate and very rapidly release preformed mediators from cytoplasmic granules, such as vasoactive amines (histamine and serotonin), proteoglycans (e.g., heparin), proteases (above all tryptases and chymases), and some pre-stored cytokines (e.g., TNFα) (1, 2, 9).

As players in innate immunity MCs have the capacity to initiate and amplify immune responses. Several lines of evidence have demonstrated that MCs participate in the sensitization phase of acquired immune responses via the secretion of mediators, which sustain dendritic cell (DC) maturation, function, and recruitment to the tissue or their migration to local draining lymph nodes (10). However, MCs also exert important effector function since MCs and T cells of different origin and subsets establish tight cell–cell interactions and modulate their respective effector functions in a bidirectional manner; this has been shown in a variety of models (11–13). Interestingly, MCs can even present antigen to T cells in a MHC class I- or class II-restricted mechanism (11, 13, 14).

**Figure 1:**
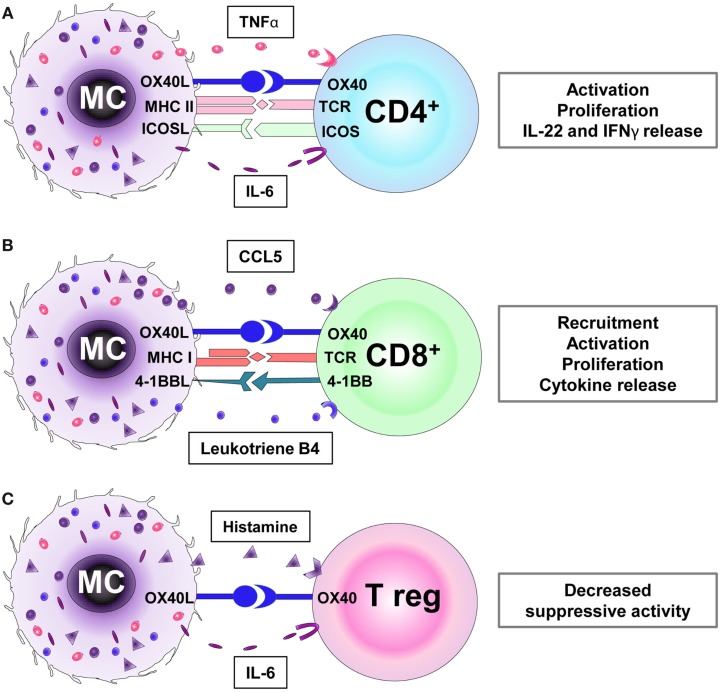
**Receptors and mediators involved in the interaction between mast cells (MCs) with CD4^+^ (A), CD8^+^ (B), and regulatory T cells (Tregs) (C)**. **(A)** MCs promote the activation, proliferation, and cytokine secretion (e.g., IL-22, IFNγ) of CD4^+^ T cells via MHC II and OX40L cell–cell interactions and TNFα secretion. **(B)** MCs induce CD8^+^ T cell recruitment via the release of chemokines (e.g., CCL5) and leukotriene B4. Furthermore, MC-mediated CD8^+^ T cell activation requires MHC I/TCR, OX40L/OX40, and 4-1BBL/4-1BB receptors interaction. **(C)** The OX40/OX40L-directed interaction between Treg and MCs and the histamine and IL-6 production by the latter inhibit the suppressive Treg activity.

This review focuses on MC-mediated regulation of T cell responses (Figure 1) since this activity not only shows MCs to be an important element of acquired immunity but also to play a cardinal role in shaping, controlling, sustaining, or arresting inflammatory responses at host–environment interfaces and, thus, of major clinical relevance.

## MCs as Regulators of CD4^+^ T Cell Effector Functions

Historically, MCs have been associated with the regulation of Th2 immune responses, and as such their modulatory activities on CD4^+^ T cells have been amply documented in many different models (Figure 1A).

In 1993, the Mecheri group reported that murine bone marrow-derived mast cells (BMMCs) displayed antigen-presenting cell (APC) functions (15), with these findings later extended to MCs of rat and human origin (16–18). Efficient BMMC antigen presentation to CD4^+^ T cells was shown to require expression of the costimulatory molecules CD80 and CD86, which are induced by IL-4 and granulocyte/macrophage-colony-stimulating factor (GM-CSF). Interestingly, in their studies, interferon (IFN) γ completely abrogated this phenomenon (19, 20); this IFNγ effect could be counteracted by FcεRI-mediated antigen endocytosis (21). In contrast to the above study, IFNγ-primed mouse MCs in their antigen-mediated interaction with CD4^+^ T cells were shown to develop a functional immunological synapse (22).

More recently, Gaudenzio and colleagues (23) have defined MCs as “tissue-localized” APCs, which (in inflamed human psoriatic skin) are primed by locally produced IFNγ to present antigen to experienced and recruited CD4^+^ T cells. IFNγ-primed human MCs establish synaptic contacts with effector/memory CD4^+^ T cells, thus inducing Th22 and IL-22^+^IFNγ^+^Th cell subsets via the release of IL-6 and TNFα. Interestingly, in inflammatory conditions in which both MCs and T cells are enriched, as seen in psoriatic skin, the majority of IL-22^+^ and IFNγ^+^CD4^+^ T cells are in close contact with MCs and the latter act as amplifiers of inflammation (23).

Since IL-6 and TNFα are mediators commonly released by activated MCs upon a wide range of stimuli, it remains unclear whether the IFNγ-induced MC search for immune partners is broad or restricted to a specific cell type or T cell subset; and how this encounter is spatially and temporally controlled. Furthermore, whether this cognate interaction leads to bidirectional effector functions, which might shape long-term MC activities is yet to be defined.

The antigen presentation activity of murine MCs and the MC-dependent modulation of effector T cell functions correlates with the induced expression of MHC class II molecules (14, 24) together with the up-regulation of a wide variety of costimulatory molecules, including members of the B7 family (ICOS ligand, PD-L1, and PD-L2) and the TNF/TNFR families (OX40L, CD153, Fas, and 4-1BB) (25).

In conjunction with the secretion of TNFα, the up-regulation of the costimulatory molecule OX40L, in particular, has been demonstrated to be essential for the MC-CD4^+^ T cell crosstalk and modulation of effector T cell function (25). OX40L expression was reported to be induced by exposure of mouse MCs to stimuli, such as toll-like receptor (TLR) agonists and FcεRI engagement (24). Furthermore, Notch signaling was shown to upregulate MHC class II and OX40L expression on mouse MCs thus promoting the proliferation CD4^+^ T cells and their differentiation into T helper 2 cells producing IL-4, IL-5, IL-10, and IL-13 (26).

Interestingly, treatment of human MCs with type I IFNs had the opposite effect of down-regulating both TNFα and OX40L expression while inducing IL-10 and TGFβ production with the consequence of restraining CD4^+^ T cell effector activities (27). This latter report underlies the key role of the inflammatory microenvironment in tightly controlling the outcome of MC–T cell interactions, and also suggests that the antigen presentation ability of MCs is possibly not intrinsic to this cell type but varies in response to time and location.

Gong and colleagues have proposed that the antigen-presenting property is restricted to an FcεRI^hi^, MHC II^+^, and c-kit^+^ mouse MC subset (28). However, considering the plasticity of MCs, one could interpret the FcεRI^hi^ and MHC II^+^ expression on MCs rather as a transitory “activation” state born of environmental (allergen) or inflammatory pressure rather than as a bona fide subset of MCs.

*In vitro* as well as *in vivo* MCs are a heterogeneous cell population, and their MHC class II expression is variable and inducible. However, MHC class II molecules are not confined to a “professional” MHC class II compartment as it is found in professional APCs, but are stored in mature and immature forms in both lysosomal and secretory granules of MCs [Ref. (29); reviewed in Ref. (11)]. Furthermore, it has been reported that MC-mediated T cell activation is mediated via exosome release (30). It is therefore tempting to speculate that antigen presentation in MCs may be the result of both direct cognate cell–cell interactions between MCs and T cells and MC-secreted MHC class II and costimulatory molecule-loaded exosomes acting upon T cells.

## MCs as Modulators of CD8^+^ T Cell Responses

Recent evidence has suggested a protective role for MCs in antiviral immune responses (31–35). This is based on the observation that MCs are equipped with a full repertoire of pattern recognition receptors, including TLRs (36), which allow MCs to sense and respond to most microbial components, including viruses.

Upon TLR engagement, mouse MCs are activated to secrete chemokines, of which notably CCL5 can recruit effector CD8^+^ T cells (37). Reovirus-infected human MCs have been shown, through release of chemokines including CCL3, CCL4, and CCL5, to selectively recruit cytotoxic effector cells, thus suggesting their ability to enhance viral immunity (31).

Furthermore, in a model of murine cytomegalovirus (CMV) infection, activated MCs have been described to recruit CD8^+^ T cells to the lungs via CCL5 release and thus contribute to a reduction in the viral load and the clearance of infection (34). MC activation upon CMV infection is characterized by an immediate TLR3/TRIF signaling-dependent phase and a delayed TLR3/TRIF-independent pathway phase (38). In allergy models, the MC-mediated recruitment of effector, but not central memory, CD8^+^ T cells to sites of inflammation was shown to be dependent on the production of MC leukotriene B4 (39).

However, the interactions between MCs and CD8^+^ T cells go far beyond that of chemokine-induced recruitment (Figure 1B). MCs have been reported to be capable of antigen presentation via MHC class I molecules to T cells following phagocytosis and processing of bacterial antigens from live bacteria (40). Furthermore, physical MC/CD8^+^ T-cell contacts have been demonstrated in healthy human skin. In lesional skin from alopecia areata (AA) patients, MCs display an activated phenotype prominently expressing MHC class I and the costimulatory molecules OX40L and 4-1BBL. Furthermore, abnormal MC numbers, effector functions, and increased interactions with CD8^+^ T cells were observed in the grafted C3H/HeJ mouse model of AA and in a recently developed humanized mouse model for AA (41). Here, in a pathological inflammatory environment, activated MCs may contribute to the collapse of hair follicle immune privilege by initiating/sustaining CD8^+^ T cell effector functions, thus promoting the disease (41).

Importantly, MC initiated antigen-dependent and MHC class I-mediated cross-presentation to CD8^+^ T cells has been shown to regulate CD8^+^ T cell effector functions including proliferation, cytokine secretion, and cytotoxic activity *in vitro*; this was supported by complementary *in vivo* studies in which antigen-specific CD8^+^ T cell numbers were reduced in MC-deficient mice, using the experimental autoimmune encephalomyelitis (EAE) model (42). These studies support previously published evidence that MC-deficient mice not only display defective CD4^+^ but also CD8^+^ T cell numbers in EAE (43) as well as in *Leishmania major* infection (44).

A specific priming of CD8^+^ effector T cells in the tissue at the site of inflammation, delivered by resident immune cells, such as MCs, may also be a relevant strategy not only to, initially, promote protective inflammation but also to control and limit excessive and/or chronic cytotoxic activity. However, very little evidence has been published to date on CD8^+^ T cell/MC interactions. Therefore, closing this important gap in our understanding of MCs functions in health and disease should be a prime future research focus.

## MCs as Suppressors of T Cell Effector Functions

Mast cells are also able to suppress T cell effector functions, namely, by their interaction with regulatory T cells (Treg) (Figure 1C). Adoptive transfer of Tregs in a mouse model of sepsis correlated with increased MC numbers (45). Furthermore, MCs contribute to the induction of tolerance to alloantigens being recruited to skin allografts in response to IL-9 secreted by Tregs (46, 47).

In line with the previously reported finding that high-FcεRI expression correlates with efficient antigen-presenting abilities in MCs (24, 28), Treg cells down-regulate FcεRI expression in MCs (48). Mouse MCs have been shown to secrete histamine and IL-6 and to use the OX40/OX40L signaling pathway to inhibit Treg functions and to thus promote optimal activation of effector CD4^+^ and CD8^+^ T cells (49–52).

However, it remains unclear which conditions promote the suppression of MC functions by Tregs versus the inhibition of Tregs by MCs. Moreover, it is conceivable that, under some conditions, MC activation may overcome Treg-mediated immunosuppression, promote the development of effective antitumor immunity, and boost the immune response in the tissue, while a different signaling environment may contribute to allograft tolerance in transplantation. Only a better definition of the relevant molecular check points will clarify the mechanisms that underlie these opposite functional outcomes and will identify promising targets for therapeutic interventions.

## Controversies in the Field

However, it should be acknowledged that the regulatory impact of MCs on T cell functions is still a controversially debated field. Namely, studies utilizing various MC-deficient mouse models have claimed that MCs are non-essential for the regulation of either CD4^+^ or CD8^+^ T cell immune responses (53, 54). Yet, this does not necessarily exclude T cell-regulatory MC activities under physiological conditions.

Mast cell function has been classically studied using the MC-deficient C57BL/6-Kit^W-sh/W-sh^ or *Kit*^W/W-V^ mice, whose MC deficiencies arise through loss of function mutations affecting Kit. However, these mice are limited in their usefulness by their perturbed immune cell composition, as such a number of new “Kit-independent” MC-deficient strains have been generated (53, 55, 56). These mice have the great advantage of deleting the MC population without apparently affecting other immune populations, with the exception of basophils (strain dependent), and have called into question findings originally obtained using Kit-dependent MC-deficient mice.

Owing to the use of multiple mouse strains and diseases models, the role of MCs in autoimmune diseases has been very controversial, with some authors tending to conclude that MCs are generally dispensable in autoimmunity (53). However, very recently, Schubert and colleagues investigated in more detail the function of MCs in arthritis using different strains of MC-deficient mice and in models, either based on autoreactive antibody transfer or effector T cells (57). Interestingly, these authors found MCs to be critically relevant in the T cell-dependent mouse model of rheumatoid arthritis [collagen-induced arthritis (CIA)], while being dispensable in the T cell-independent antibody-induced arthritis model. In the CIA model, absence of MCs resulted in dramatic loss of T cell expansion upon immunization and concomitant reduction in T cell cytokine responses (57). These recent findings underscore the critical role of MCs in T cell-dependent autoimmunity.

However, in a T cell-dependent spontaneous diabetes model, using non-obese diabetic (NOD) mice, MCs failed to impact on CD4^+^ and CD8^+^ T cell numbers measured at the onset of the disease (54). However, this study left it unclear whether at later time points during disease progression, i.e., when the phenotype divergence between MC deficient and wild-type mice may be greatest, or during spontaneous disease resolution, the absence or presence of MCs would have impacted upon T cell responses and clinical outcome.

Possibly, the most contentious issue has been the role of MCs in multiple sclerosis (MS), particularly in the T cell-dependent surrogate mouse model of EAE, with some studies arguing an important role for MCs (58, 59), while another one claims that they are dispensable (53). This controversial discussion has been very important and productive in the sense that it has brought to light the limitations in the use of each of the presently available MC-deficient mouse strains, and has underscored the urgent need for standardized disease-induction protocols to improve data reproducibility. Furthermore, these discrepancies have served to acutely remind us of the constitutive difficulties one faces in translating murine data to the human condition.

## Conclusion and Perspectives

In this review, we have highlighted interactions between MCs and T cells, which regulate adaptive immune responses and have delineated that the antigen-presenting activity of tissue-resident immune cells, such as MCs, is fundamental to the maintenance of productive and protective inflammation. MCs may also actively participate in the fundamental processes, which minimize immune-mediated bystander damage to healthy tissues.

We have also reviewed the evidence that MC can modulate Treg activities. However, the mechanisms and dynamics that interrupt MC-mediated antigen presentation, down-regulate the MC-induced amplification of T cell-dependent immune response, and restore local Treg cell homeostasis, all remain to be dissected by future research.

This review has closed with discussing contradictory results and the ensuing controversial debate on the role of MC in experimental autoimmune disease. It is important to keep in mind that the conflicting findings were generated using different MC-deficient mouse strains. This raises the pertinent question whether models, which rely on the deletion of an entire cell population, such as MCs, which are notoriously heterogeneous, highly plastic and adaptable in nature, and excel in their capacity to rapidly shift the spectrum of mediators released and surface markers expressed in distinct signaling environments (e.g., homeostatic versus inflammatory settings), are not overly simplistic. Can such models possibly reflect the (very transitory) dynamics of MC biology *in vivo*? Therefore, the ultimate research tool for definitively clarifying the contribution of MCs to the regulation of T cell functions under physiological and pathological conditions, which fully takes into account the dynamism and heterogeneity of MCs, may still have to be developed.

## Author Contributions

SP and RB drafted and revised the manuscript.

## Conflict of Interest Statement

The authors declare that the research was conducted in the absence of any commercial or financial relationships that could be construed as a potential conflict of interest.
